# Correction: Factors associated with COVID-19 vaccine uptake and hesitancy among healthcare workers in the Democratic Republic of the Congo

**DOI:** 10.1371/journal.pgph.0005096

**Published:** 2025-08-20

**Authors:** Michel K. Nzaji, Jean de Dieu Kamenga, Christophe Luhata Lungayo, Aime Cikomola Mwana Bene, Shanice Fezeu Meyou, Anselme Manyong Kapit, Alanna S. Fogarty, Dana Sessoms, Pia D. M. MacDonald, Claire J. Standley, Kristen B. Stolka

The fifth author’s initials appear incorrectly in the citation. The correct citation is: Nzaji MK, Kamenga JdD, Lungayo CL, Bene ACM, Fezeu Meyou S, Kapit AM, et al. (2024) Factors associated with COVID-19 vaccine uptake and hesitancy among healthcare workers in the Democratic Republic of the Congo. PLOS Glob Public Health 4(2): e0002772. https://doi.org/10.1371/journal.pgph.0002772.
