# Supplementary material for: Factors associated with COVID-19 vaccine uptake and hesitancy among healthcare workers in the Democratic Republic of the Congo
Source: PLOS Glob Public Health. 2024 Feb 1;4(2):e0002772. doi: 10.1371/journal.pgph.0002772 (PMC10833569; doi:10.1371/journal.pgph.0002772)
Supplement: S1 Table — Percentages are calculated across rows. Reference variable noted in the OR column. OR = odds ratio; CI = confidence interval. (DOCX) [file pgph.0002772.s001.docx]

**Table S1. Univariate regression analyses of factors significantly associated with COVID-19 vaccine status (N = 5,102)**.

| **Factor** | **Number (%)** | **Vaccinated**  **n (%)** | **Unvaccinated**  **n (%)** | **OR (CI 95%)** | ***P*-value** |
| --- | --- | --- | --- | --- | --- |
| **Age range** | | | | | |
| 18-55 | 4658 (91.3%) | 2089 (44.8%) | 2569 (55.2%) | Reference |  |
| >55 | 444 (8.7%) | 275 (61.9%) | 169 (38.1%) | 2.00 (1.64-2.44) | <.001 |
| **Sex** |  |  |  |  |  |
| Female | 2565 (50.27%) | 1070(41.7%) | 1495 (58.3%) | Reference |  |
| Male | 2537 (49.73%) | 1294 (51.0%) | 1243 (49.0%) | 1.46 (1.30-1.62) | <.001 |
| **Province** | | | | | |
| Kasai Oriental | 550 (10.78%) | 408 (74.2%) | 142 (25.8%) | Reference |  |
| Kinshasa | 900 (17.64%) | 382 (42.4%) | 618 (56.6%) | 0.22 (0.17-0.27) | <.001 |
| Haut Katanga | 832 (16.3%) | 355 (42.7%) | 477 (57.3%) | 0.26 (0.21-0.33) | <.001 |
| Kongo Central | 896 (17.56%) | 347 (38.7%) | 549 (61.3%) | 0.22 (0.17-0.28) | <.001 |
| Lualaba | 591 (11.58%) | 268 (45.3%) | 323 (54.7%) | 0.29 (0.23-0.38) | <.001 |
| North Kivu | 422 (8.27%) | 138 (32.7%) | 284 (67.3%) | 0.17 (0.13-0.22) | <.001 |
| South Kivu | 911 (17.86%) | 466 (51.2%) | 445 (48.8%) | 0.37 (0.30-0.47) | <.001 |
| **Healthcare worker categories** | | | | | |
| Nurse | 2356 (46.18%) | 1122 (47.6%) | 1234 (52.4%) | Reference |  |
| Doctor | 814 (15.95%) | 423 (52.0%) | 391 (48.0%) | 1.18 (1.01-1.93) | .032 |
| Pharmacist | 208 (4.08%) | 81 (38.9%) | 127 (61.1%) | 0.70 (0.52-0.94) | <.001 |
| Midwife | 444 (8.7%) | 191 (43.0%) | 253 (56.1%) | 0.83 (0.68-1.01) | .074 |
| Laboratory technician | 462 (9.05%) | 203 (43.9%) | 259 (56.1%) | 0.82 (0.70-1.05) | .147 |
| Other | 818 (16.03%) | 344 (42.1%) | 474 (57.9%) | 0.79 (0.68-0.93) | .006 |
| **Marital status** | | | | | |
| Married | 3540 (69.9%) | 1810 (51.1%) | 1730 (48.9%) | Reference |  |
| Single | 926 (18.15%) | 277 (29.9%) | 649 (70.1%) | 0.40 (0.34-0.47) | <.001 |
| Divorce/Separated | 90 (1.76%) | 44 (48.9%) | 46 (51.1%) | 0.91 (0.60-1.38) | .674 |
| Cohabitation | 376 (7.37%) | 153 (40.7%) | 223 (59.3%) | 0.65 (0.52-0.81) | <.001 |
| Widowed | 170 (3.33%) | 80 (47.1%) | 90 (52.9%) | 0.84 (0.62-1.15) | .299 |
| **Place of residence** | | | | | |
| Urban | 4488 (87.96%) | 1996 (44.5%) | 2492 (55.5%) | Reference |  |
| Rural | 614 (12.04%) | 368 (59.9%) | 246 (40.1%) | 1.87 (1.57-2.22) | <.001 |
| **Other vaccine uptake** | | | | | |
| No | 3137 (61.5%) | 1318 (42.0%) | 1819 (58.0%) | Reference |  |
| Yes | 1965 (38.5%) | 1046 (53.2%) | 919 (46.8%) | 1.57 (1.40-1.76) | <.001 |
| **Existing chronic illness** | | | | | |
| No and don’t know | 4540 (88.99%) | 2052 (45.2%) | 2488 (54.8%) | Reference |  |
| Yes | 562 (11.01%) | 312 (55.5%) | 250 (44.5%) | 1.51 (1.29-1. 81) | <.001 |
| **Perceived risk of contracting COVID-19** | | | | | |
| No risk | 368 (7.21%) | 163 (44.3%) | 205 (55.7%) | Reference |  |
| Low | 862 (16.89%) | 327 (37.9%) | 535 (62.1%) | 0.77 (0.60-0.99) | .036 |
| Moderate | 2149 (42.12%) | 956 (44.5%) | 1193 (55.5%) | 1.01 (0.81-1.26) | .945 |
| High | 1723 (33.77%) | 918 (53.3%) | 805 (46.7%) | 1.43 (1.14-1.80) | <.001 |
| **Previously tested for COVID-19** | | | | | |
| Yes | 2216 (43.43%) | 1143 (51.6%) | 1073 (48.4%) | Reference |  |
| No | 2886 (56.56%) | 1221 (42.3%) | 1665 (57.7%) | 1.45 (1.30-1.62) | <.001 |
| **Knowledge of availability of different COVID-19 vaccines in province** | | | | | |
| Yes | 4577 (89.7%) | 2237 (48.9%) | 2340 (51.1%) | Reference |  |
| No | 525 (10.3%) | 127 (24.2%) | 398 (75.8%) | 0.33 (0.27-0.41) | <.001 |
| **Awareness of routine vaccination against COVID-19 in province or local area** | | | | | |
| Yes | 4163 (81.59%) | 2051 (49.3%) | 2112 (50.7%) | Reference |  |
| No | 939 (18.4%) | 313 (33.3%) | 626 (66.7%) | 0.51 (0.44-0.59) | <.001 |
| **Aware of the planned vaccination campaign against COVID-19 in province or local area** | | | | | |
| Yes | 4745 (93.0%) | 2226 (46.9%) | 2519 (53.1%) | Reference |  |
| No | 357 (7.0 %) | 138 (38.7%) | 219 (61.3%) | 0.18 (0.15-0.21) | <.001 |
| **Vaccination within respondent’s facility** | | | | | |
| Yes | 3827 (75.0%) | 1977 (51.7%) | 1850 (48.3%) | Reference |  |
| No | 1275 (25.0%) | 387 (30.4%) | 888 (69.6%) | 0.40 (0.35-0.46) | <.001 |
| **Previous work at a COVID-19 vaccination site** | | | | | |
| Yes | 1540 (30.2%) | 990 (64.3%) | 550 (35.7%) | Reference |  |
| No | 3562 (69.8%) | 1374 (38.6%) | 2188 (61.4%) | 0.34 (0.30-0.39) | <.001 |
| **Willingness to take a COVID-19 vaccination if available in the province** | | | | | |
| Yes | 3430 (67.2%) | 2290 (66.8%) | 1140 (33.2%) | Reference |  |
| No | 1672 (32.8%) | 74 (4.4%) | 1598 (95.6%) | 0.02 (0.01-0.02) | <.001 |

Percentages are calculated across rows. Reference variable noted in the OR column. OR = odds ratio; CI = confidence interval.
