# Supplementary material for: Factors associated with COVID-19 vaccine uptake and hesitancy among healthcare workers in the Democratic Republic of the Congo
Source: PLOS Glob Public Health. 2024 Feb 1;4(2):e0002772. doi: 10.1371/journal.pgph.0002772 (PMC10833569; doi:10.1371/journal.pgph.0002772)
Supplement: S2 Table — Responses were recorded on a Likert scale, with 1 the least level of agreement and 5 the strongest level of agreement. A response of 3 was described as “partially agree”. (DOCX) [file pgph.0002772.s002.docx]

**Table S2. Decisions influencing COVID-19 vaccination, by vaccination status.**

| **Statement related to factors that might influence or incentivize vaccination** | **Vaccinated (n=2,364)** | **Unvaccinated (n=2,738)** | ***P*-value** |
| --- | --- | --- | --- |
|  | **Mean (SD)** | **Mean (SD)** |  |
| **If I were convinced that getting vaccinated would help protect vulnerable members of my family or community.** | 3.21 (1.36) | 2.62 (1.47) | <.001 |
| **If I were sure that the vaccine is effective and that people who are vaccinated do not get sick with COVID-19.** | 3.04 (1.41) | 2.75 (1.50) | <.001 |
| **If someone I knew got sick, was hospitalized, died from COVID-19.** | 2.67 (1.34) | 2.15 (1.23) | <.001 |
| **If I believe there will soon be new drugs to treat COVID-19 infection.** | 2.60 (1.36) | 2.12 (1.34) | <.001 |
| **If getting vaccinated was a requirement for my job.** | 2.51 (1.40) | 2.22 (1.32) | <.001 |
| **If colleagues or family members encouraged me to get vaccinated.** | 2.50 (1.32) | 1.98 (1.18) | <.001 |
| **If I believe that I am healthy and can resist infection with COVID-19.** | 2.49 (1.31) | 2.05 (1.24) | <.001 |
| **If my spouse asks me to get vaccinated.** | 2.48 (1.35) | 1.99 (1.19) | <.001 |
| **If my religious leaders said I should get vaccinated.** | 2.43 (1.32) | 1.96 (1.19) | <.001 |
| **If we were given food each vaccination session.** | 1.66 (1.00) | 1.46 (0.84) | <.001 |
| **If I were to receive a financial incentive.** | 1.64 (1.04) | 1.46 (0.88) | <.001 |

Responses were recorded on a Likert scale, with 1 the least level of agreement and 5 the strongest level of agreement. A response of 3 was described as “partially agree”.
