# Supplementary material for: Factors associated with COVID-19 vaccine uptake and hesitancy among healthcare workers in the Democratic Republic of the Congo
Source: PLOS Glob Public Health. 2024 Feb 1;4(2):e0002772. doi: 10.1371/journal.pgph.0002772 (PMC10833569; doi:10.1371/journal.pgph.0002772)
Supplement: S3 Table — Responses were recorded on a Likert scale, with 1 the least level of agreement and 5 the strongest level of agreement. A response of 3 was described as “partially agree”. (DOCX) [file pgph.0002772.s003.docx]

**Table S3.** **Participant’s level of confidence and social trust in government authorities in the fight against COVID-19, by vaccination status.**

|  | **Vaccinated** | **Unvaccinated** | ***P*-value** |
| --- | --- | --- | --- |
|  | **n = 2,364** | **n = 2,738** |  |
| **Statement** | Mean (SD) | Mean (SD) |  |
| **I trust the authorities in the fight against COVID-19.** | 3.76 (0.97) | 3.31 (1.29) | <.0001 |
| **I trust the information provided by the media in the fight against COVID-19.** | 3.71 (0.97) | 3.26 (1.23) | <.0001 |
| **I trust in our health system and our hospitals in the fight against COVID-19.** | 3.75 (0.98) | 3.36 (1.18) | <.0001 |
| **I trust that the government’s actions in the fight against COVID-19 are the right ones.** | 3.60 (1.0) | 3.12 (1.20) | <.0001 |
| **I trust that the measures to combat COVID-19 will be properly implemented.** | 3.62 (0.95) | 3.15 (1.17) | <.0001 |
| **I trust in the relevance of the economic measures taken vis-à-vis COVID-19.** | 3.24 (1.18) | 2.80 (1.30) | <.0001 |
| **I think we are more successful in the fight against COVID-19 than western countries in the fight against COVID-19.** | 3.41 (1.20) | 3.15 (1.34) | <.0001 |

Responses were recorded on a Likert scale, with 1 the least level of agreement and 5 the strongest level of agreement. A response of 3 was described as “partially agree”.
