# Supplementary material for: Factors associated with COVID-19 vaccine uptake and hesitancy among healthcare workers in the Democratic Republic of the Congo
Source: PLOS Glob Public Health. 2024 Feb 1;4(2):e0002772. doi: 10.1371/journal.pgph.0002772 (PMC10833569; doi:10.1371/journal.pgph.0002772)
Supplement: S1 File — (DOCX) [file pgph.0002772.s004.docx]

**File S1. Knowledge, Attitudes, Practices Questionnaire (in French and *English*)**

**Acceptabilité de la vaccination COVID-19 chez le personnel de santé en RDC**

Les agents de santé sont un groupe à haut risque de contracter le COVID-19 en raison de leurs interactions avec les patients, c'est pourquoi la vaccination contre la COVID-19 a été identifiée comme une stratégie clé pour les protéger. Cette étude vise à examiner l'expérience COVID-19, la perception de la lutte contre le COVID-19 et l'acceptation du vaccin COVID-19 parmi les agents de santé en RDC. Ceci est purement à des fins de recherche et que votre participation est volontaire et votre anonymat est assuré. Merci d'avoir accepté de participer à cette enquête.

***Acceptability of COVID-19 vaccination among health workers in the DRC***

*Healthcare workers are a high-risk group for contracting COVID-19 due to their interactions with patients, therefore vaccination against COVID-19 has been identified as a key strategy to protect them. This study aims to examine the COVID-19 experience, the perception of the fight against COVID-19 and the acceptance of the COVID-19 vaccine among health workers in the DRC. This is purely for research purposes and your participation is voluntary and your anonymity is assured.*

*Thank you for agreeing to participate in this survey.*

1. **Caractéristiques sociodémographiques**

***Socio-demographic characteristics***

1. Quel âge avez-vous?

*How old are you ?*

1. Quel est votre sexe ?
2. Masculin
3. Féminin

*What is your gender?*

1. *Male*
2. *Female*
3. Quel est votre appartenance religieuse?
4. Chrétien
5. Musulman
6. Animiste
7. Sans religion
8. Autre précisez):­­­­­­­­­­­­­­­­­­­­­­­­­­________________

*What is your religious affiliation?*

1. *Christian*
2. *Muslim*
3. *Animist*
4. *No religion*
5. *Other (specify):­­­­­­­­­­­­­­­­­­­­­­­­­­ ________________*
6. Quel est votre niveau d’étude ?
7. Primaire
8. Secondaire
9. Supérieur/ou universitaire
10. N’a pas étudié

*What is your level of education?*

1. *Primary*
2. *Secondary*
3. *Higher education and/or university*
4. *Did not study*
5. A quelle catégorie d'agents de santé appartenez-vous ?

Infirmier(e)

Sage-femme

Technicien(ne) de laboratoire

Pharmacien(ne)

- 1. Médecin
  2. Autre précisez):________________

*What category of health worker do you belong to?*

*Nurse*

*Midwife*

*Laboratory Technician*

*Pharmacist*

1. *Doctor*
2. *Other (specify):________________*
3. Quel est votre état civil actuel ?
4. Marié
5. Union libre
6. Divorcé (e) / séparé
7. Veuf (ve)
8. Celibataire

*What is your current marital status?*

*1. Married*

*2. Common-law union*

*3. Divorced (e) / separated*

*4. Widow(er)*

*5. Single*

1. Dans quelle province vous résidez ? :

*In which province do you reside? :*

Nom de la zone de sante :

*Name of the health zone:*

Nom de la structure :

*Name of the structure :*

1. Lieu de résidence:
2. Urbain
3. Rural

*Place of residence:*

1. *Urban*
2. *Rural*
3. En dehors de la vaccination dans l’enfance et de celle contre la COVID-19, avez-vous déjà reçu d’autres vaccins ?
4. Oui
5. Non

*Apart from the childhood vaccination and the COVID-19 vaccination, have you ever received any other vaccines?*

*1. Yes*

*2. No*

Si Non, passez à la question 12.

*If No, go to question 12*.

1. Si oui, quel type de vaccination avez-vous reçu ?
2. Vaccin contre le cholera
3. Vaccin contre la fièvre jaune
4. Vaccine contre la maladie à virus Ebola
5. Autres (à spécifier)

*If yes, what type of vaccination did you receive?*

1. *Cholera vaccine*
2. *Yellow fever vaccine*
3. *Vaccine against Ebola virus disease*
4. *Other (please specify)*
5. A quand remonte cette vaccination en mois ?

*When did this vaccination take place in months?*

1. Avez-vous une maladie chronique?
2. Oui
3. Non
4. Je ne sais pas

*Do you have a chronic illness?*

1. *Yes*
2. *No*
3. *I do not know*
4. **Expérience COVID-19**
5. Pensez-vous que vous pouvez contracter la maladie à Coronavirus ?
6. Oui, comme tout le monde
7. Oui, j'ai plus de risque que d'autres personnes
8. Non
9. Ne sait pas

*Do you think you can get the Coronavirus disease?*

*1. Yes, like everyone else*

*2. Yes, I am more at risk than other people*

*3. No*

*4. Don't know*

1. Avez-vous déjà été en contact avec un patient positif au COVID-19 ?
2. Oui
3. Non
4. Ne sait pas

*Have you ever been in contact with a COVID-19 positive patient?*

*1. Yes*

*2. No*

*3. Don't know*

Si autres réponses que OUI, passez à 16.

*If other than YES, go to 16.*

1. Si oui, qu’avez-vous fait ?
2. Rien
3. Effectuer un examen de laboratoire
4. Prendre un traitement contre la COVID-19
5. Autres (à spécifier)

*If so, what did you do?*

1. *Nothing*
2. *Perform a laboratory examination*
3. *Take treatment for COVID-19*
4. *Other (please specify)*
5. Avez-vous déjà fait un examen pour le diagnostic de COVID-19?
6. Oui
7. Non

*Have you ever been tested for COVID-19?*

*1. Yes*

*2. No*

Si non, passez à 19.

*If not, go to 19.*

1. Si oui, quel a été le résultat du test ?
2. Positif
3. Négatif
4. Je n'ai pas reçu le résultat
5. Je ne sais pas

*If so, what was the result of the test?*

1. *Positive*
2. *Negative*
3. *I did not receive the result*
4. *I don't know*
5. A quand remonte votre dernier test COVID-19 en mois ?

*When was you last COVID-19 test in months?*

1. Quelle est votre risque de contracter le COVID-19 ?
2. Petit
3. Modéré
4. Elevé
5. Aucun risque

*What is your risk of contracting COVID-19?*

1. *Small*
2. *Moderate*
3. *High*
4. *No risk*

**Veuillez indiquer dans quelle mesure vous êtes d'accord ou en désaccord avec chaque affirmation sur la confiance sociale dans la lutte contre la pandémie de Covid-19 en RDC**

***Please indicate the extent to which you agree or disagree with each statement about social trust in the fight against the Covid-19 pandemic in DRC***

**Cochez *Check***

|  |  | **Pas du tout d'accord** | **Être en désaccord** | **Partiellement d'accord** | **D'accord** | **Tout à fait d'accord** |
| --- | --- | --- | --- | --- | --- | --- |
|  | Je fais confiance aux autorités dans la lutte contre le Covid-19 |  |  |  |  |  |
|  | Je fais confiance aux informations fournies par les médias dans la lutte contre le Covid-19 |  |  |  |  |  |
|  | J'ai confiance en notre système de santé et nos hôpitaux dans la lutte contre le Covid-19 |  |  |  |  |  |
|  | Je fais confiance à la justesse des mesures prises par le gouvernement dans la lutte contre le Covid-19 |  |  |  |  |  |
|  | Je fais confiance à la bonne mise en œuvre des mesures de lutte contre le Covid-19 |  |  |  |  |  |
|  | J'ai confiance dans la pertinence des mesures économiques prises vis-à-vis du Covid-19 |  |  |  |  |  |
|  | Je pense que nous réussissons mieux dans la lutte contre le Covid-19 que les pays occidentaux dans la lutte contre le Covid-19 |  |  |  |  |  |
|  | Je pense que la pandémie de Covid-19 va perdre son effet avec le soleil |  |  |  |  |  |
|  | Je pense que la pandémie de Covid-19 va perdre son effet avec nos produits traditionnels |  |  |  |  |  |
|  | Je pense que la pandémie de Covid-19 va perdre son effet avec la vaccination |  |  |  |  |  |
|  | Le Covid-19 a montré que les pays réputés puissants ne sont pas si puissants |  |  |  |  |  |

|  |  | ***Strongly Disagree*** | ***Disagree*** | ***Partially Agree*** | ***Agree*** | ***Strongly Agree*** |
| --- | --- | --- | --- | --- | --- | --- |
| 20. | *I trust the authorities in the fight against Covid-19* |  |  |  |  |  |
| 21. | *I trust the information provided by the media in the fight against Covid-19* |  |  |  |  |  |
| 22. | *I have confidence in our health system and our hospitals in the fight against Covid-19* |  |  |  |  |  |
| 23. | *I have confidence that the government’s actions in the fight against Covid-19 are the right ones* |  |  |  |  |  |
| 24. | *I trust that the measures to combat Covid-19 will be properly implemented.* |  |  |  |  |  |
| 25. | *I have confidence in the relevance of the economic measures taken vis-à-vis Covid-19* |  |  |  |  |  |
| 26. | *I think we are more successful in the fight against Covid-19 than western countries in the fight against Covid-19* |  |  |  |  |  |
| 27. | *I think the Covid-19 pandemic will lose its effect with the sun* |  |  |  |  |  |
| 28. | *I think the Covid-19 pandemic will lose its effect with our traditional products* |  |  |  |  |  |
| 29. | *I think the Covid-19 pandemic will lose its effect with vaccination* |  |  |  |  |  |
| 30. | *Covid-19 has shown that countries deemed powerful are not so powerful* |  |  |  |  |  |

1. **Intention de se faire vacciner**

***Intention to vaccinate***

1. Avez-vous entendu parler de la vaccination en routine contre le COVID-19 dans votre province / commune / Quartier ou village ?
2. Oui
3. Non

*Have you heard about routine vaccination against COVID-19 in your province/township/neighborhood or village?*

1. *Yes*
2. *No*
3. Avez-vous entendu parler de la campagne de vaccination prévue contre le COVID-19 dans votre province / commune / Quartier ou village ?
   - - 1. Oui
       2. Non

*Have you heard about the planned vaccination campaign against COVID-19 in your province / commune / neighborhood or village?*

*1. Yes*

*2. No*

1. Si oui, par quel canal avez-vous eu cette information ?
2. Récos /CAC
3. Membres de familles ou communautés
4. Formations sanitaires ou agents de santé
5. Radio
6. Télévision
7. Eglise
8. Ecole
9. Réseaux sociaux
10. Affiches / panneaux / banderoles / flyers/ etc….
11. Autres

*If so, how did you get this information?*

1. *Recos/CAC*
2. *Family or community members*
3. *Health facilities or health workers*
4. *Radio*
5. *Television*
6. *Church*
7. *School*
8. *Social networks*
9. *Posters / billboards / banners / flyers / etc….*
10. *Others*
11. Saviez-vous que plusieurs vaccins contre la COVID-19 sont présents dans votre province / commune / quartier ou village / sites de vaccination, le prendriez-vous ?
12. Oui
13. Non

*Did you know that several vaccines against COVID-19 are present in your province / municipality / district or village / vaccination sites, would you take it?*

*1. Yes*

*2. No*

1. Avez-vous déjà été vacciné contre la COVID-19?
2. Oui, une dose
3. Oui, deux doses
4. Non

*Have you ever been vaccinated against COVID- 19?*

1. *Yes, one dose*
2. *Yes, two doses*
3. *No*

Si non passez à 38.

*If not go to 38.*

1. Si oui, quelles ont été les motivations pour accepter le vaccin contre la COVID-19?
2. Pour se protéger et protéger les autres
3. Croyance en la vaccination et en la science
4. Pour aider à arrêter la propagation du virus
5. Pour revenir à la vie d’avant (sans masque, sans distanciation sociale, etc…)
6. Pour faciliter mon voyage
7. Pour ne pas mourir
8. Autres à spécifier

*If yes, what were the motivations for accepting the COVID- 19 vaccine?*

1. *To protect yourself and others*
2. *Belief in vaccination and science*
3. *To help stop the spread of the virus*
4. *To return to life before (without mask, without social distancing, etc.)*
5. *To facilitate my trip*
6. *To not die*
7. *Others to be specified*
8. Si oui, parmi les différents types de vaccins présents dans votre province, lequel vous a été administré ?
9. Moderna
10. Pfizer
11. Sinovac
12. Astrazeneca
13. Johnson and Johnson
14. Autres (à préciser)

*If yes, among the different types of vaccines in your province, which one were administered to you?*

1. *Moderna*
2. *Pfizer*
3. *Sinovac*
4. *Astrazeneca*
5. *Johnson and Johnson*
6. *Other (s) to be specified)*
7. Quelles sont les facteurs ayant influencé le choix du vaccin reçu ?
8. Effets indésirables
9. Durée de protection
10. Seuil d'efficacité
11. Pays d’origine du vaccin
12. Nombre de doses d’administration
13. Technologie des vaccins à ARNm
14. Connaissance des cas de décès après vaccination

*What factors influenced the choice of vaccine received?*

1. *Side effects*
2. *Duration of protection*
3. *Efficiency threshold*
4. *Country of origin of the vaccine*
5. *Number of administration doses*
6. *mRNA vaccine technology*
7. *Knowledge of cases of death after vaccination*
8. Si non, quelle est la principale raison de votre refus de recevoir le vaccin COVID-19?
9. Données insuffisantes sur l'innocuité du nouveau vaccin
10. Préoccuper par les effets indésirables du vaccin
11. Une inquiétude sur l'inefficacité du vaccin
12. Réaction indésirable antérieure à tout vaccin
13. Je suis contre les vaccins en général
14. Une préoccupation d'acquérir une infection au COVID-19 à partir du vaccin lui-même
15. Je me perçois comme ne présentant pas un risque considérable de développer des complications si je suis infecté par COVID-19
16. Je ne me perçois pas à risque élevé de contracter une infection au COVID-19
17. J'ai déjà eu une infection au COVID-19
18. L'administration du vaccin est douloureuse ou incommode
19. Pas confiance à cause du délai court de la fabrication des vaccins
20. A cause du plan des occidentaux ou les illuminaties d’éliminer les africains à travers les vaccins
21. La protection de Dieu suffit, pas besoin de vaccin.
22. Le vaccin rend stérile
23. Autre (à préciser) : __________________________

*If not, what is the main reason for your refusal to receive the COVID- 19 vaccine?*

*1. Insufficient data on the safety of the new vaccine*

*2. Concern about vaccine side effects*

*3. Concern about the ineffectiveness of the vaccine*

*4. Previous adverse reaction to any vaccine*

*5. I am against vaccines in general*

*6. A concern of acquiring COVID-19 infection from the vaccine itself*

*7. I perceive myself as not being at considerable risk of developing complications if I become infected with COVID-19*

*8. I do not perceive myself to be at high risk of contracting a COVID-19 infection*

*9. I have had a COVID-19 infection before*

*10. Vaccine administration is painful or inconvenient*

*11. Lack of confidence because of the short lead time for the manufacture of vaccines*

*12. Because of the plan of the Westerners or the Illuminati to eliminate Africans through vaccines*

*13. God's protection is enough, no vaccine needed.*

*14. The vaccine makes you sterile*

*15. Other (specify) : __________________________*

1. Veuillez indiquer si les éléments suivants pourraient avoir une influence sur votre décision de vous faire vacciner :

|  |  | **Pas du tout d'accord** | **Être en désaccord** | **Partiellement d'accord** | **D'accord** | **Tout à fait d'accord** |
| --- | --- | --- | --- | --- | --- | --- |
| 1 | Si je devais recevoir une incitation financière |  |  |  |  |  |
| 2 | Si on donnerait à manger à chaque séance de la vaccination |  |  |  |  |  |
| 3 | Si me faire vacciner était une exigence pour mon travail |  |  |  |  |  |
| 4 | Si je crois que je suis en bonne santé et que je peux résister à une infection au COVID-19 |  |  |  |  |  |
| 5 | Si j'étais convaincu que me faire vacciner aiderait à protéger les membres vulnérables de ma famille ou de ma communauté |  |  |  |  |  |
| 6 | Si quelqu'un que je connaissais est tombé malade, a été hospitalisé, est décédé de COVID-19 |  |  |  |  |  |
| 7 | Si des collègues ou des membres de ma famille m'ont encouragé à me faire vacciner |  |  |  |  |  |
| 8 | Si mes chefs religieux disaient que je devrais me faire vacciner |  |  |  |  |  |
| 9 | Si j'étais sûr que le vaccin est efficace et que les personnes vaccinées ne tombent pas malades avec COVID-19 |  |  |  |  |  |
| 10 | Si mon (ma) conjoint (e) me demande de me faire vacciner |  |  |  |  |  |
| 11 | Si je crois qu'il y aura bientôt de nouveaux médicaments pour traiter l'infection à COVID-19 |  |  |  |  |  |

*Please indicate whether the following might influence your decision to be vaccinated.*

|  |  | ***Strongly Disagree*** | ***Disagree*** | ***Partially Agree*** | ***Agree*** | ***Strongly Agree*** |
| --- | --- | --- | --- | --- | --- | --- |
| *1* | *If I were to receive a financial incentive* |  |  |  |  |  |
| *2* | *If we would give food at each vaccination session* |  |  |  |  |  |
| *3* | *If getting vaccinated was a requirement for my job* |  |  |  |  |  |
| *4* | *If I believe I am healthy and can withstand COVID-19 infection* |  |  |  |  |  |
| *5* | *If I believed that getting vaccinated would help protect vulnerable members of my family or community* |  |  |  |  |  |
| *6* | *If someone I knew got sick, was hospitalized, died of COVID-19* |  |  |  |  |  |
| *7* | *If colleagues or family members encouraged me to get vaccinated* |  |  |  |  |  |
| *8* | *If my religious leaders said I should get vaccinated* |  |  |  |  |  |
| *9* | *If I was sure that the vaccine is effective and that vaccinated people do not get sick with COVID-19* |  |  |  |  |  |
| *10* | *If my spouse asks me to get vaccinated* |  |  |  |  |  |
| *11* | *If I believe there will soon be new drugs to treat COVID-19 infection* |  |  |  |  |  |
